# Intensity-Modulated Radiation Therapy for Rectal Carcinoma Can Reduce Treatment Breaks and Emergency Department Visits

**DOI:** 10.1155/2012/891067

**Published:** 2012-08-13

**Authors:** Salma K. Jabbour, Shyamal Patel, Joseph M. Herman, Aaron Wild, Suneel N. Nagda, Taghrid Altoos, Ahmet Tunceroglu, Nilofer Azad, Susan Gearheart, Rebecca A. Moss, Elizabeth Poplin, Lydia L. Levinson, Ravi A. Chandra, Dirk F. Moore, Chunxia Chen, Bruce G. Haffty, Richard Tuli

**Affiliations:** ^1^Department of Radiation Oncology, The Cancer Institute of New Jersey, UMDNJ-Robert Wood Johnson Medical School, 195 Little Albany Street, New Brunswick, NJ 08903, USA; ^2^Department of Radiation Oncology, Albert Einstein Medical Center, New York, NY 10467, USA; ^3^Department of Radiation Oncology and Molecular Radiation Sciences, Johns Hopkins University School of Medicine, Baltimore, MD 21231, USA; ^4^Department of Radiation Oncology, Loyola University, Maywood, IL 60153, USA; ^5^Department of Medical Oncology, Johns Hopkins University School of Medicine, Baltimore, MD 21231, USA; ^6^Department of Surgical Oncology, Johns Hopkins University School of Medicine, Baltimore, MD 21231, USA; ^7^Division of Medical Oncology, The Cancer Institute of New Jersey, UMDNJ-Robert Wood Johnson Medical School, 195 Little Albany Street, New Brunswick, NJ 08903, USA; ^8^Department of Radiation Oncology, University of Virginia Medical Center, Charlottesville, VA 22908, USA; ^9^Department of Biostatistics, The Cancer Institute of New Jersey, UMDNJ-Robert Wood Johnson Medical School, 195 Little Albany Street, New Brunswick, NJ 08903, USA; ^10^Department of Radiation Oncology, Cedars Sinai Medical Center, Los Angeles, CA 90048, USA

## Abstract

*Purpose*. To compare the acute toxicities of IMRT to 3D-conformal radiation therapy (3DCRT) in the treatment of rectal cancer. *Methods and Materials*. Eighty-six patients with rectal cancer preoperatively treated with IMRT (*n* = 30) and 3DCRT (*n* = 56) were retrospectively reviewed. Rates of acute toxicity between IMRT and 3DCRT were compared for anorexia, dehydration, diarrhea, nausea, vomiting, weight loss, radiation dermatitis, fatigue, pain, urinary frequency, and blood counts. Fisher's exact test and chi-square analysis were applied to detect statistical differences in incidences of toxicity between these two groups of patients. *Results*. There were fewer hospitalizations and emergency department visits in the group treated with IMRT compared with 3DCRT (*P* = 0.005) and no treatment breaks with IMRT compared to 20% with 3DCRT (*P* = 0.0002). Patients treated with IMRT had a significant reduction in grade ≥3 toxicities versus grade ≤2 toxicities (*P* = 0.016) when compared to 3DCRT. The incidence of grade ≥3 diarrhea was 9% among 3DCRT patients compared to 3% among IMRT patients (*P* = 0.31). *Conclusions*. IMRT for rectal cancer can reduce treatment breaks, emergency department visits, hospitalizations, and all grade ≥3 toxicities compared to 3DCRT. Further evaluation and followup is warranted to determine late toxicities and long-term results of IMRT.

## 1. Introduction

The standard management of locally advanced rectal cancer consists of preoperative 5-fluorouracil (5-FU)-based chemoradiation (CRT), which has been established over the past several decades with multiple pivotal clinical trials. A large randomized trial compared preoperative to postoperative CRT for locally advanced rectal cancer and demonstrated that neoadjuvant treatment improved local control and reduced toxicity [1]. Although both short- and long-term side effects decreased with a preoperative approach, absolute rates of toxicity were still noteworthy. Rates of grade 3-4 diarrhea were 12% with preoperative CRT and 18% in the postoperative setting. All acute grade 3-4 toxicities (diarrhea, hematologic, and dermatologic) were 27% with preoperative chemoradiation versus 40% with postoperative therapy.

Since intensity-modulated radiation therapy (IMRT) has the potential to improve dose distributions to nearby dose-limiting structures, it is of potential benefit in the management of rectal cancer with a recent study showing a reduction in gastrointestinal toxicity [2]. It may help reduce dose to bowel, bone marrow, and bladder and therefore reduces the associated organ-specific side effects for cervical, prostate, and anal cancers. For carcinoma of the cervix, pelvic IMRT permitted sparing of pelvic bone marrow [3] and was associated with lower toxicity rates and favorable outcomes compared to standard radiation therapy [4]. Additionally, for prostate cancer patients treated with androgen deprivation therapy, IMRT significantly reduced acute and late GI toxicities compared to 3DCRT [5]. For anal canal carcinoma, IMRT appeared comparable to 3DCRT with regard to local control and survival while decreasing dermatologic, GI, and hematological toxicities and associated treatment breaks [6, 7].

For the management of rectal cancer, dosimetric studies have shown that IMRT reduces doses of irradiated small bowel [8]. This study seeks to evaluate the toxicity profiles and clinical data with IMRT versus 3DCRT for rectal cancer, with the hypothesis that IMRT would lessen the severity of acute toxicities during the preoperative management of rectal cancer.

## 2. Materials and Methods 

Under a protocol approved by the institutional review boards (IRB) of three institutions, patients with rectal cancer treated with concurrent CRT were identified. The procedures followed were in accordance with the IRB ethical standards and with the Helsinki Declaration of 1975, as revised in 2000. Preoperative CRT was the preferred paradigm among patients with locally advanced rectal cancer. Postoperative radiation therapy cases were omitted, and patients treated preoperatively with IMRT and 3DCRT were analyzed. All patients provided informed consent for treatment. Concurrent chemotherapy consisted of continuous infusion 5-fluorouracil at doses of 225 mg/m^2^ or capecitabine 825 mg/m^2^ twice a day. Other chemotherapy regimens included capecitabine 825 mg/m^2^ twice a day, concurrently with irinotecan or oxaliplatin 50 mg/m^2^ weekly and radiation therapy. Also, a regimen of oxaliplatin 130 mg/m^2^ on day 1, followed by 5-day continuous infusion 5-FU 350 mg/m^2^ and leucovorin 100 mg/m^2^ during weeks 1 and 5, was used with radiation therapy (Table 1). Patient weight, performance status, total treatment time, need for treatment breaks, and toxicity assessments were performed prior to, weekly during treatment, and 6–8 weeks after chemoradiation. The use of antidiarrheal and antiemetic medications was documented on weekly medication flow sheets. Any use of intravenous fluids was documented in the chart on separate physician order sheets. Toxicities were graded according to the Common Terminology Criteria for Adverse Events Version 3.0. Acute toxicities assessed included anorexia, dehydration, diarrhea, nausea, vomiting, weight loss, radiation dermatitis, fatigue, pain, and urinary frequency. Blood counts were also assessed. Hospital admissions and emergency department visits were available via the electronic medical record for all institutions. The data on treatment breaks was extracted from the record and verify system of each institution. The rates of toxicities among patients receiving IMRT were then compared to those treated with 3DCRT. Fischer's exact test was applied to test for statistically significant side effects related to treatment with IMRT and 3DCRT. A value of *P* ≤ 0.05 was considered significant.

### 2.1. Radiation Therapy Planning

Computed tomography (CT)-based simulation with 2.5 mm slice thickness was performed. Patients were simulated either supine or prone (IMRT, 97% supine) (3DCRT, 91% prone) with arms up and a full bladder. Oral contrast was given to patients for small bowel delineation. A custom immobilization was designed for supine patients, and a belly board was used for those placed in the prone position.

Gross tumor volume (GTV) and enlarged regional lymph nodes were determined by a combination of findings on physical exam, transrectal ultrasound, CT, PET-CT, and/or MRI. The clinical target volume (CTV) was defined as the GTV plus internal iliac (T3) and external iliac (T4) and perirectal, mesorectal, and presacral lymph nodes. The rectal CTV included the rectal GTV with a 1.5–2 cm radial expansion and 2.5–3 cm craniocaudal expansion, while the nodal GTV was given a 1.5–2 cm uniform expansion. Uninvolved iliac nodal regions had a 1.0–1.5 cm expansion. The presacral lymph nodes began at the sacral promontory and ended at the bottom of S5. CTV and mesorectum were generated according to the RTOG anorectal contouring atlas when available [9]. PTV expansions were 0.5–1.0 cm.

### 2.2. IMRT Technique

**Figure 1:**
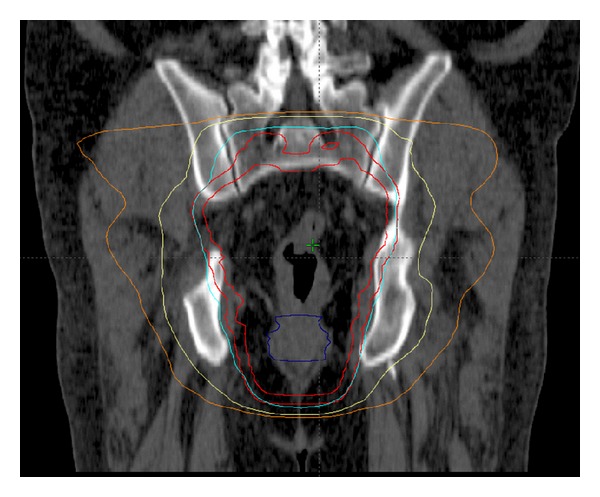
Coronal images of an IMRT plan.

**Figure 2:**
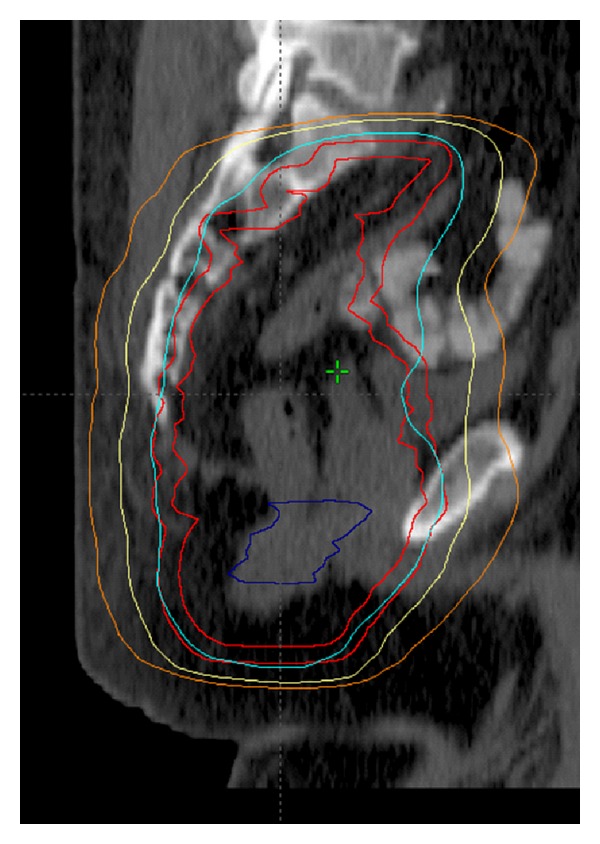
Sagittal images of an IMRT plan. This 56-year-old man was found to have an uT3N0 rectal adenocarcinoma and managed with preoperative CRT consisting of capecitabine with a seven-field coplanar IMRT plan to a total dose of 4500 cGy followed by a boost to the GTV to a total of 5040 cGy. The 95%, 70%, and 50% isodose curves are displayed along with the PTV.

A total of 4500 cGy in 180 cGy daily fractions was delivered to the pelvis (rectum and draining lymph nodes at risk) using inverse-planned IMRT. This was followed by a cone-down phase consisting of either a 3-dimensional conformal boost designed with a 3-field technique to GTV and a minimum 2 cm uniform margin including all of the presacral space for an additional 540 cGy in 180 cGy daily fractions or an IMRT plan with the same volumes. Every effort was made to limit the dose to the small and large bowel doses. Figures 1 and 2 demonstrate a representative IMRT plan. Three radiation oncologists, who specialize in the management of gastrointestinal malignancies, prepared the field design of these cases. Prior to incorporation of these IMRT cases in this series, each submitting radiation oncologist reviewed their cases to ensure that they met the abovementioned planning constraints.

Small bowel, femoral head, and bladder IMRT constraints were followed as per RTOG 0822. For patients treated prior to release of RTOG 0822 (*n* = 1), patients whose plans met the RTOG 0822 constraints were included in this analysis. Inverse planning with seven-to-nine equally spaced, coplanar IMRT fields was constructed. Image guidance with either cone-beam CT or orthogonal films was utilized daily. Acute toxicities were defined as those which occurred during or up to 8 weeks following the completion of CRT.

## 3. Results

All study patients with rectal cancer were treated from 2005 to 2011, while IMRT was utilized after 2007. Thirty patients (35%) received IMRT, and 56 patients (65%) received 3DCRT to a median total dose of 5040 cGy (IMRT range 5000–5040 cGy and 3DCRT range 4500 cGy–5140 cGy). There were no significant differences in median age, gender, type or duration of chemotherapy, or stage between groups (Table 1). Median followup time was 23 months in the 3DCRT group compared to 11 months in the IMRT group (*P* = 0.00005).

Patients who received IMRT had a significant reduction in all grade ≥3 toxicities versus grade ≤2 toxicities (*P* = 0.016) with respect to hematological, urinary, pain, fatigue and GI (anorexia, dehydration, diarrhea, nausea, vomiting, and weight loss) symptoms, as compared to those treated with 3DCRT (Table 2). The rate of GI grade ≥3 (*n* = 9 with 3DCRT versus *n* = 1 with IMRT) toxicities versus grade ≤2 toxicities was not significantly different (*P* = 0.085). The incidence of grade ≥3 diarrhea was 9% among 3DCRT patients compared to 3% among IMRT patients (*P* = 0.31).

Overall, patients who received two or more chemotherapy agents concurrently with radiation therapy demonstrated higher rates of grade ≥3 toxicity (43%) compared to those receiving single-agent chemotherapy (11%; *P* = 0.009). On multivariate analysis, there was a significant relationship between GI toxicity and chemotherapy type when adjusting for age and stage with the two or more chemotherapy group having higher toxicity than the one chemotherapy type group when treated with either 3DCRT or IMRT (*P* = 0.022). On multivariate analyses, there were no significant relationships found among the use of infusional 5-FU or capecitabine and the outcomes of pathological complete response, GI toxicity, or all toxicity.

There were fewer hospitalizations and emergency department visits in the group treated with IMRT (2%) compared with 3DCRT (14%; *P* = 0.005). There were no treatment breaks with IMRT compared to 20% with 3DCRT (*P* = 0.0002). Likewise, the median total time to treatment completion was shorter with IMRT (38.7 days) versus 3DCRT (40.5 days) but did not reach statistical significance (*P* = 0.081). From the start to end of radiation therapy, performance status showed less decline in the IMRT group with 33% of patients showing a decline in performance status and 51% in the 3DCRT group (*P* = 0.091).

### 3.1. Pathologic Response Rates

The rates of pathological complete response were similar in the 3DCRT group at 21% versus 20% with IMRT (*P* = 0.55). Preoperative tumor T-stage downstaging was similar with 50% of 3DCRT patients and 60% of IMRT patients (*P* = 0.25). Nodal downstaging occurred in 34% of 3DCRT patients and 40% of IMRT patients (*P* = 0.37). Also, rates of local recurrence were similar between the groups with 6.7% local failure in the IMRT group and 7% in the 3DCRT group (*P* = 0.65). Of the local failures in the IMRT group, none were marginal failures. Rates of distant metastases after completion of therapy were not significantly different at 12.5% of 3DCRT patients and 6.7% of IMRT patients (*P* = 0.33).

## 4. Discussion

By implementing inverse planning and improving conformality of targets, IMRT allows limitation of radiation dose to nearby normal organs at risk, while allowing delivery of high doses to the tumor and regional lymph nodes. In so doing, it can reduce side effects by conforming dose to avoid normal, uninvolved tissues, which may correlate with an improvement in the toxicity profile. The use of IMRT for rectal cancer may also potentially prevent delays in time to surgery, facilitate improved postoperative healing, and allow improved tolerability of adjuvant chemotherapy [2]. This is the first study to show that IMRT not only results in a more timely administration of chemoradiation, but also results in fewer hospitalizations and emergency room visits.

IMRT for rectal cancer can reduce treatment-related toxicities, as compared to standard 3DCRT. In our study, IMRT significantly reduced all toxicities including GI, hematological, urinary, pain, and fatigue, compared to 3DCRT. Toxicity management is well enumerated in patient charts at these tertiary care hospitals, with significant documentation of prescribed medications according to JCAHO regulations, which are accounted in the CTCAE grading. In this study, grades of diarrhea were lessened but overall GI toxicities (independent of hematological, urinary, pain, and fatigue) were not significantly reduced with IMRT compared to 3DCRT.

Patients tolerated IMRT with fewer treatment breaks relative to 3D-CRT. Despite the omission of the postoperative patients, treatment breaks still remained at a level of 20% in the 3D-CRT cohort. Although the possibility of hospitalizations/ED visits and treatment breaks could be due to chance, the use of the electronic record to determine elapsed treatment days and hospitalization/ED visits increases the objectivity of this measure.

Patient characteristics were quite similar between the groups with regard to age, stage, and chemotherapies used. Although there was no prospective quality assurance of the plans, all IMRT planning was conducted by only three radiation oncologists (SKJ, JMH, SNN) with expertise and primary focus in the management gastrointestinal malignancies. The RTOG guidelines and anorectal contouring atlas were employed. Although the median followup was not long, assessment of short-term toxicity was the main endpoint.

Understanding the limitations of a retrospective comparison, the rates of toxicity seen with IMRT in this series appear encouraging when evaluated in relation to prior studies. The 3DCRT toxicity rates were comparable to the German rectal trial in terms of diarrhea, with a rate of grade ≥3 diarrhea of 12–18% compared to 9% in our 3DCRT group and 3% in the IMRT group [1]. The NSABP R-03 trial which randomized rectal cancer patients to preoperative (with one cycle of induction 5-FU and leucovorin before chemoradiation) or postoperative RT (3DCRT) with concurrent 5-FU and leucovorin showed a rate of 36% of grade ≥3 diarrhea for the preoperative arm and 29% for the postoperative group [10].

A prior dosimetric comparison of 3DCRT to IMRT for rectal cancer showed that the bowel volume irradiated was significantly reduced with IMRT [11]. Specifically, the planning techniques most successful at bowel sparing were a 3-field forward planned IMRT technique and a 9-field equally spaced IMRT technique [11]. In addition, IMRT can reduce median doses to small bowel by 5.1 Gy for rectal cancer [8]. Other studies have demonstrated improvements in target coverage, homogeneity, and conformality, while reducing doses to the small bowel, bladder, and pelvic bones for preoperatively planned cases in the prone position [12]. Likewise, implementation of IMRT and CT-based image-guidance can decrease irradiated small bowel and the normal tissue complication probability [13]. Therefore, the data suggest that the volume of small bowel irradiated can be reduced with IMRT.

Patients who received IMRT in our series were usually treated in the supine position to improve the setup reproducibility and tolerability, whereas most of the patients who received 3DCRT were treated in the prone position. One study evaluating the optimal method for reducing irradiated small bowel volumes in preoperative rectal cancer patients showed that a combination of prone positioning with bladder distention was most effective [14], but this was in an Asian population with presumably smaller body habitudes than Americans. In contrast, another study showed no difference in toxicity outcomes with the use of IMRT in prone versus supine positioning for endometrial cancer [15]. Drzymala et al. compared supine versus prone position in 19 rectal cancer patients and showed that at the low dose levels, a significantly higher volume of bowel was irradiated in the supine position, but from 20 to 45 Gy, there was no significant difference in the volume of bowel irradiated with each 5 Gy increment. Therefore, the volume of bowel irradiated at doses associated with bowel toxicity with concurrent CRT was not significantly higher in the supine position [16]. The data as to the optimal positioning of patients for pelvic RT is conflicting, and the benefit of bowel sparing with each of these techniques (bladder distention, positioning, IMRT) may be incremental or patient dependent and requires further study. 

Another important consideration with the use of IMRT is the potential for compromising outcomes by missing or potentially underdosing tumor and target volumes. In our study, the efficacy of IMRT downstaging appeared to be similar to 3DCRT. Of the small cohort of patients treated with IMRT, only two experienced local recurrence, neither of which were marginal failures. However, further followup is needed to adequately assess outcome, and this rate was not significantly different compared to patients treated with 3D-CRT. Given the potential for marginal failures with the use of IMRT for rectal cancer, care must be taken to contour according to available data and atlases. Prior studies in other GI malignancies have shown the importance of adherence to protocol in order to achieve the expected benefit of radiation therapy [17].

Certainly, daily changes in organ positioning can potentially impact outcomes, including side effects. However, with the available information from anal cancer IMRT and cervical cancer IMRT, we recognize that the benefit of these treatments was realized even in the setting of daily changes in positioning. In this study, interfraction motion was corrected by the use of on-board imaging. In our study, patients were instructed and counseled on appropriate bladder filling and rectal emptying procedures. In addition, expansions for GTV to CTV and PTV were quite reasonably sized to achieve PTV's in IMRT planning that resembled those of 3DCRT fields. In fact, with the expansions used in our study, the PTV received full dose, whereas using standard field arrangements, the IMRT PTV often would have been in the penumbra. This situation occurs because standard 3DCRT blocks are usually placed 2 cm from the iliac vessels therefore, full dose is delivered approximately 1–1.2 cm from the iliac vessels due to normal dose falloff. However, in our IMRT expansions, these normal iliac vessels would have been expanded 1.0–1.5 cm, with an additional margin of 0.5–1.0 cm for PTV, which would then receive full dose with IMRT to the PTV.

It should be noted that two of the four grade-3 leukopenia cases in the 3DCRT arm occurred in patients who were treated with concurrent oxaliplatin, leucovorin, and 5-FU. Nevertheless, the other two patients who experienced grade-3 leukopenia received capecitabine alone with radiation therapy. There was no change in toxicity with the administration of 5-day versus 7-day 5-FU or capecitabine, and no differences were detected between 5-FU and capecitabine in terms of outcomes. Due to the self-administration of capecitabine, patient adherence to medication administration could be a factor in determining patient outcomes. 

In this cohort, chemotherapy was primarily 5-FU or capecitabine concurrently with radiation. The available literature about this topic has been quite clear that the standard of care is 5-day continuous infusion 5 fluorouracil or 5 days of capecitabine concurrent with radiation therapy. The NSABP R-04 trial demonstrated no differences in outcomes including pathological complete response rates, surgical downstaging, or sphincter sparing surgery with either of these regimens [18]. Given that the majority of our patients were treated with concurrent 5-FU or capecitabine, the cohorts appear relatively homogeneous and comparable.

Our study demonstrates that IMRT may help reduce treatment interruptions, emergency department visits, and hospitalizations compared to 3DCRT. In addition, grade ≥3 toxicities were rare in this IMRT cohort. Grade ≥3 diarrhea was also reduced with the use of IMRT compared to 3DCRT. This series adds to the available literature favoring the use of IMRT in gastrointestinal malignancies. However, additional studies are needed to assess the impact of IMRT on long-term clinical outcomes and late toxicities in the treatment of rectal adenocarcinoma.

## Figures and Tables

**Table 1:** Patient characteristics.

Characteristic	3DCRT (*n* = 56)	IMRT (*n* = 30)	*P* value
Mean age (years)	56.3	52.7	0.23
Male/female (*n*) (%)	40/16 (71/29)	15/15 (50/50)	0.06
Total elapsed days (mean)	40.5	38.7	0.081
Treatment suspended (*n*) (%)	11 (20%)	0 (0)	0.0002
Median total dose (cGy)	5040	5040	0.23

Chemotherapy types (*n*) (%)			0.71
Capecitabine or 5-FU	45 (80)	27 (90)	
Capecitabine/oxaliplatin	7(12)	3 (10)	
Capecitabine/CPT-11	1 (2)	0	
5-FU/leucovorin/oxaliplatin	3 (5)	0	

Preoperative T stage (*n*) (%)			0.85
T2	0	1 (3)	
T3	51 (91)	27 (90)	
T4	5 (9)	2 (7)	

Preoperative N stage (*n*) (%)			0.21
N0/x	26 (46)	10 (33)	
N1/N2	30 (54)	20 (67)	

Preoperative M stage (*n*)%			0.91
M0	52 (93)	27 (90)	
M1	4 (7)	3 (10)	

Pathological complete response (%)	21%	20%	0.555
Downstaging T stage (%)	50	60	0.26
Downstaging N stage (%)	34	40	0.37

**Table 2:** Rates of toxicity by CTCAE grade.

Toxicity	3DCRT *n* (%)	IMRT *n* (%)	*P*
Diarrhea			0.31
Grade 0–2	51 (91)	29 (97)	
Grade 3-4	5 (9)	1 (3)	

Dehydration			0.17
Grade 0–2	52 (93)	30 (100)	
Grade 3-4	4 (7)	0	

Nausea/vomiting			
Grade 0–2	56 (100)	30 (100)	
Grade 3-4	0	0	

Fatigue			
Grade 0–2	56 (100)	30 (100)	
Grade 3-4	0	0	

Pain			0.72
Grade 0–2	54 (96)	29 (97)	
Grade 3-4	2 (4)	1 (3)	

Urinary frequency			
Grade 0–2	55 (98)	30 (100)	0.65
Grade 3-4	1 (2)	0	

Nadir white blood cell count			0.27
Grade 0–2	53 (95)	30 (100)	
Grade 3-4	3 (5)	0	

Nadir Hemoglobin			0.42
Grade 0–2	54 (96)	30 (100)	
Grade 3-4	2 (4)	0	
